# PROTOCOLS FOR COLLECTING CONVERSATIONAL LANGUAGE IN PERSONS WITH DEMENTIA

**DOI:** 10.1093/geroni/igad104.2415

**Published:** 2023-12-21

**Authors:** Oleg Zaslavsky, Trevor Cohen, Kuan-Ching Wu, Jessica Jin, Shao-Yun Chien, Serguei Pakhomov

**Affiliations:** University of Washington, Seattle, Washington, United States; University of Washington, Seattle, Washington, United States; University of Washington, Seattle, Washington, United States; University of Washington, Seattle, Washington, United States; University of Washington, Seattle, Washington, United States; University of Minnesota, Minneapolis, Minnesota, United States

## Abstract

We describe first-of-its-kind protocols for collecting spontaneous conversations in persons with dementia living in a community through conversation elicitation tools twice a month over six months. We also describe the preliminary feasibility of the protocol deployment in the first enrolled participants over the first month of data collection. Participants were recruited from the University of Washington ADRC registry. The audio was captured using RODE wireless microphone system. Four conversational elicitation techniques were used: 1) reminiscence using personal photographs; 2) reminiscence using generic historical images; 3) picture description test from neurocognitive batteries; 4) open-ended questions about daily activities. Health measures included Cornell Scale for Depression in Dementia (CSDD), Clinical Dementia Rating (CDR), and Clinical Frailty Scale (CFS). Four male and two female participants (average age 75) were recruited. Average health measures at baseline were CDR = 2, suggesting moderate dementia; CSDD=7, suggesting depressive symptoms, CFS=5, suggesting mild frailty. At baseline, the tasks’ average conversation time in seconds was as follows: daily activities =528; personal reminiscence=443; historical reminiscence =227; picture description =192. The corresponding average times for the subsequent home visits were 917; 518; 474, and 280 at visit 1 and 525; 767; 629, and 311 at visit 2. We established the feasibility of collecting conversational speech in this population. Participants seemed more interested in conversations focused on daily activities and personal pictures than general topics. There was an increase in the duration of conversations over time, which might be related to participants’ greater familiarity with the study protocol and data collectors.

